# Increased Frequency of Dysfunctional Siglec-7^−^CD57^+^PD-1^+^ Natural Killer Cells in Patients With Non-alcoholic Fatty Liver Disease

**DOI:** 10.3389/fimmu.2021.603133

**Published:** 2021-02-22

**Authors:** Yuzuru Sakamoto, Sachiyo Yoshio, Hiroyoshi Doi, Taizo Mori, Michitaka Matsuda, Hironari Kawai, Tomonari Shimagaki, Shiori Yoshikawa, Yoshihiko Aoki, Yosuke Osawa, Yuji Yoshida, Taeang Arai, Norio Itokawa, Masanori Atsukawa, Takanori Ito, Takashi Honda, Yoshihiro Mise, Yoshihiro Ono, Yu Takahashi, Akio Saiura, Akinobu Taketomi, Tatsuya Kanto

**Affiliations:** ^1^Department of Liver Diseases, The Research Center for Hepatitis and Immunology, National Center for Global Health and Medicine, Tokyo, Japan; ^2^Department of Gastoenterological Surgery I, Hokkaido University Graduate School of Medicine, Sapporo, Japan; ^3^Division of Gastroenterology and Hepatology, Department of Internal Medicine, Nippon Medical School, Tokyo, Japan; ^4^Division of Gastroenterology and Hepatology, Nagoya University Graduate School of Medicine, Nagoya, Japan; ^5^Department of Hepato-Pancreatic-Biliary Surgery, Japanese Foundation for Cancer Research, Tokyo, Japan

**Keywords:** mass cytometry (CyTOF), Siglec, NK cell, PD-1, CD57, NAFLD

## Abstract

Non-alcoholic fatty liver disease (NAFLD) is a progressive disorder that can develop into liver fibrosis and hepatocellular carcinoma. Natural killer (NK) cells have been shown to protect against liver fibrosis and tumorigenesis, suggesting that they may also play a role in the pathogenesis of NAFLD. Sialic acid-binding immunoglobulin-like lectins (Siglecs) are a family of inhibitory and activating receptors expressed by many cell types, including NK cells. Here, we investigated the phenotypic profiles of peripheral blood and intrahepatic NK cells, including expression of Siglecs and immune checkpoint molecules, and their association with NK cell function in patients with NAFLD. Immune cells in the peripheral blood of 42 patients with biopsy-proven NAFLD and 13 healthy volunteers (HVs) were identified by mass cytometry. The function of various NK cell subpopulations was assessed by flow cytometric detection of intracellular IFN-γ and CD107a/LAMP-1, a degranulation marker, after *in vitro* stimulation. We found that peripheral blood from NAFLD patients, regardless of fibrosis stage, contained significantly fewer total CD56^+^ NK cell and CD56^dim^ NK cell populations compared with HVs, and the CD56^dim^ cells from NAFLD patients were functionally impaired. Among the Siglecs examined, NK cells predominantly expressed Siglec-7 and Siglec-9, and both the expression levels of Siglec-7 and Siglec-9 on NK cells and the frequencies of Siglec-7^+^CD56^dim^ NK cells were reduced in NAFLD patients. Notably, Siglec-7 levels on CD56^dim^ NK cells were inversely correlated with PD-1, CD57, and ILT2 levels and positively correlated with NKp30 and NKp46 levels. Further subtyping of NK cells identified a highly dysfunctional Siglec-7^−^CD57^+^PD-1^+^CD56^dim^ NK cell subset that was increased in patients with NAFLD, even those with mild liver fibrosis. Intrahepatic NK cells from NAFLD patients expressed elevated levels of NKG2D and CD69, suggesting a more activated phenotype than normal liver NK cells. These data identify a close association between NK cell function and expression of Siglec-7, CD57, and PD-1 that could potentially be therapeutically targeted in NAFLD.

## Introduction

Non-alcoholic fatty liver disease (NAFLD) is a heterogenous group of diseases characterized by fat accumulation in the liver. The prevalence and public health burden of NAFLD is increasing worldwide (1), as illustrated by the situation in Asia, where the current population prevalence of NAFLD is about 25% (2). Like other chronic liver diseases, NAFLD can progress to fibrosis, cirrhosis, and hepatocellular carcinoma (HCC) in some patients. The degree of fibrosis is independently associated with long-term overall mortality in NAFLD patients (3). Therefore, identifying the drivers of liver fibrosis and inhibiting its progression to cirrhosis and HCC is a crucial step in improving the prognosis of NAFLD patients.

Natural killer (NK) cells are critical components of the innate immune system and play vital roles in producing cytokines and chemokines and in directly killing aberrant cells, including virally infected and tumor cells. The cytotoxic activity of NK cells is induced by receptor-mediated engagement of target cells and the release of granules containing cytotoxic proteins such as granzyme and perforin (4). The production of interferon-γ (IFN-γ) by NK cells also enhances the cytotoxic activity of NK cells and cytotoxic T lymphocytes against tumor cells. Such direct and indirect anti-tumor activities highlight the importance of NK cells in preventing and controlling malignant diseases (5).

NK cells are reported to play an additional role in liver fibrosis, where they are thought to reduce fibrogenesis by killing activated hepatic stellate cells or by altering the phenotype of liver macrophages (6, 7). Indeed, reductions in the frequency and function of NK cells are observed in patients with HCC and are associated with poor prognosis (8–10). Similarly, NK cell dysfunction has also been reported in patients with viral hepatitis and alcoholic liver disease (11, 12). However, the phenotypes and functions of NK cells in NAFLD patients, and their potential contribution to the disease, are unclear.

Human NK cells can be classified into two subsets according to their expression of surface markers, such as CD56 and CD16 (FcγRIIIa). CD56^bright^CD16^−^ NK cells are potent cytokine producers and are relatively scarce, whereas CD56^dim^CD16^+^ NK cells exhibit higher cytotoxic activity and account for 90–95% of the peripheral NK cell population (13). NK cells also express an array of surface proteins that include differentiation markers (CD27, CD16, and CD57), activation markers (NKp30, NKp46, NKG2D, CD38, and CD69), and inhibitory markers (PD-1, ILT2, TIGIT, Tim-3, NKG2A, and KIRs). These molecules interact with specific soluble or cell surface ligands to regulate the function of NK cells (14). Of note, the immune checkpoint protein PD-1 is reportedly upregulated on NK cells from patients with various cancers, including HCC (15). However, little is known about the expression of such checkpoint molecules on NK cells in NAFLD.

Sialic acid-binding immunoglobulin-like lectins (Siglecs) are a family of lectins that act as cell surface receptors for molecules containing characteristic sialic acid linkages and can transmit inhibitory or activation signals into the cell (16–22). Siglecs are differentially expressed on hematopoietic cells in humans, with NK cells mainly expressing Siglec-7 and Siglec-9 (22). Although binding of specific antibodies or ligands to Siglec-7 inhibits NK cell function (23, 24), Siglec-7 downregulation is indicative of NK cell dysfunction (25, 26). Siglec-9 expression defines a subset of cytotoxic NK cells with a mature phenotype (23). Decreased Siglec-9 expression on CD56^dim^ NK cells has been reported in patients with chronic hepatitis B or malignant melanoma (23, 27). Nevertheless, the impact of Siglec-7 and Siglec-9 expression on NK cells in patients with NAFLD remains unclear.

In this study, we sought to clarify various aspects of NK cell involvement in NAFLD. We examined the phenotypic profiles of Siglecs and immune checkpoint molecules using mass cytometry, and investigated the functional relevance of NK cell subsets by examining cytokine and cytolytic granule production by flow cytometry. We found that the frequency and function of NK cells were reduced in NAFLD patients compared with healthy subjects, and the impairments were already evident in patients with mild fibrosis. We also identified an association between NAFLD and an increased frequency of a highly dysfunctional subset of Siglec-7^−^CD57^+^PD-1^+^CD56^dim^ NK cells in patients with NAFLD.

## Materials and Methods

### Study Cohorts

Written informed consent was obtained from all subjects at enrollment. The study conformed to the ethical guidelines of the 1975 Declaration of Helsinki and the ethical guidelines for human clinical research established by the Japanese Ministry of Health, Labor and Welfare. The study protocol was approved by the ethics committees of the National Center for Global Health and Medicine (NCGM-G-002355-05), Nippon Medical School (685), Nagoya University Hospital (2019-0030-15255), and the Cancer Institute Hospital of Japanese Foundation for Cancer Research (2017-1118).

Peripheral blood samples were obtained from 42 patients with liver biopsy-confirmed NAFLD who were followed at Kohnodai Hospital, Nippon Medical School Chiba Hokusoh Hospital, Abiko Toho hospital, and Nagoya University Hospital. The 42 patients consisted of 27 with no or mild fibrosis (F stage 0, 1, or 2) and 15 with advanced fibrosis (F3 or 4). As controls, peripheral blood was collected from 13 healthy volunteers (HVs) who had no apparent history of liver disease or malignancies and were negative for hepatitis B surface antigen (HBsAg), human immunodeficiency virus (HIV) antigen, anti-HIV antibodies, and anti-hepatitis C virus (HCV) antibodies. Table 1 shows the clinicopathological characteristics of the 42 NAFLD patients and 13 HVs.

**Table 1:** Clinicopathological characteristics of NAFLD patients stratified by fibrosis stage.

		**NAFLD performed liver biopsy (*****n*** **=** **42)**		***P*-value**
	**HV**	**F0-2**	**F3-4**	
	**(*n* = 13)**	**(*n* = 27)**	**(*n* = 15)**	
**Age (years)**	44.5 ± 10.8	59.2 ± 12.5	65.2 ± 10.6	***P*** **<** **0.001**
Gender (male, %)	69.2	29.6	46.7	NS
**BMI (kg/m**^**2**^**)**	21.7 ± 2.2	29.0 ± 3.8	28.6 ± 4.0	***P*** **<** **0.0001**
HT +/-	-	6 / 21	5 / 10	NS
HL +/-	-	8 / 19	4 / 11	NS
AST (IU/L)	-	46.8 ± 24.8	56.3 ± 21.8	NS
ALT (IU/L)	-	57.9 ± 31.4	58.8 ± 34.8	NS
T-bil (mg/dL)	-	1.0 ± 0.5	1.5 ± 1.4	NS
Alb (g/dL)	-	4.2 ± 0.5	3.8 ± 0.6	NS
**Plt (x10**^**4**^**/μL)**	-	21.8 ± 5.5	12.1 ± 5.1	***P*** **<** **0.0001**
**PT (%)**	-	99.6 ± 16.5	83.8 ± 21.2	***P*** **<** **0.05**
Hb (g/dL)	-	14.1 ± 1.9	13.7 ± 1.6	NS
TP (mg/dL)	-	7.1 ± 0.7	7.2 ± 0.6	NS
**T-Cho (g/dL)**	-	203.0 ± 39.2	173.1 ± 32.9	***P*** **<** **0.05**
γGTP (IU/L)	-	66.9 ± 66.4	72.8 ± 29.6	NS
ALP (IU/L)	-	260.3 ± 77.7	261.3 ± 107.2	NS
HbA1c (%)	-	6.5 ± 0.7	6.5 ± 1.1	NS
AFP (ng/mL)	-	3.8 ± 2.1	5.2 ± 3.4	NS
**M2BPGi**	-	1.6 ± 3.0	3.4 ± 3.7	***P*** **<** **0.001**
**FIB-4**	-	2.1 ± 1.7	5.2 ± 3.4	***P*** **<** **0.001**
**AAR**	-	0.9 ± 0.5	1.1 ± 0.3	***P*** **<** **0.05**
**APRI**	-	0.6 ± 0.4	1.4 ± 0.7	***P*** **<** **0.0001**
**GPR**	-	0.7 ± 0.7	1.6 ± 1.3	***P*** **<** **0.001**
ALBI	-	−2.5 ± 0.5	−2.1 ± 0.7	NS
NAS	-	3 (1–5)	4 (2–6)	NS
Steatosis	-	1 (0–2)	1 (0–2)	NS
Lobular inflammation	-	2 (0–2)	2 (1–3)	NS
Hepatocyte ballooning	-	1 (0–1)	1 (0–2)	NS

*Data are presented as patient number, mean ± SD values, or score (range) as appropriate. P-values were determined by the Kruskal–Wallis test with Dunn's multiple comparison test except for the comparison between NAFLD patients with F0–2 and F3–4, which were analyzed by the Mann–Whitney U-test. The bold values mean the significance as P-value less than 0.05*.

*AAR, aspartate aminotransferase to alanine aminotransferase ratio; AFP, alpha-fetoprotein; Alb, serum albumin; ALBI, albumin-bilirubin grade; ALP, alkaline phosphatase; ALT, alanine aminotransferase; APRI, aspartate aminotransferase to platelet ratio index; AST, aspartate aminotransferase; BMI, body mass index; FIB-4, fibrosis-4 index; F0–2, fibrosis stage 0, 1, and 2; F3–4, fibrosis stage 3 and 4; GPR, gamma-glutamyl transpeptidase to platelet ratio; Hb, hemoglobin; HbA1c, hemoglobin A1c; HT, hypertension; HL, hyperlipidemia; HV, healthy volunteer; M2BPGi, Mac-2 binding protein glycosylation isomer; NAFLD, non-alcoholic fatty liver disease; NAS, NAFLD activity score; NS, not significant; Plt, platelet counts; PT, prothrombin time; T-bil, total bilirubin; T-cho, total cholesterol; TP, total protein; γGTP, gamma-glutamyl transpeptidase*.

For direct comparison of peripheral blood NK and intrahepatic NK cells, peripheral blood samples and non-cancerous liver tissues were obtained from four patients who underwent liver resection for NAFLD-associated HCC at Kohnodai Hospital or the Cancer Institute Hospital of Japanese Foundation for Cancer Research. As controls for these experiments, peripheral blood and non-cancerous liver tissues were obtained from four patients who underwent liver resection for metastatic liver tumors. Table 2 shows the clinicopathological characteristics for these eight subjects.

**Table 2:** Clinicopathological characteristics of liver specimen donors patients.

**Patients**	**Case**	**Age**	**Gender**	**Hight (m)**	**Weight (kg)**	**BMI (kg/m^**2**^)**	**Tumor characteristics**			**Non-cancerous liver tissues**				
							**Differentiation**	**Size**	**St/Mt**	**F stage**	**NAS**	**Steatosis**	**Lobular inflammation**	**Hepatocyte ballooning**
NAFLD-HCC	1	65	M	1.66	86.3	31.2	Mod	5.2	Mt	2	1	1	0	0
	2	63	M	1.62	72.6	27.6	Mod	3	St	4	1	1	0	0
	3	75	F	1.49	47.1	21.1	Mod	4	St	3	1	0	1	0
	4	67	M	1.68	70.2	24.8	Mod	2	St	4	4	1	1	2
Liver metastasis	1	59	F	1.74	60.4	19.9	N/A	N/A	N/A	0	N/A	N/A	N/A	N/A
	2	47	F	1.62	56.6	21.5	N/A	N/A	N/A	0	N/A	N/A	N/A	N/A
	3	56	M	1.72	78	26.4	N/A	N/A	N/A	0	N/A	N/A	N/A	N/A
	4	73	M	1.71	56.3	19.3	N/A	N/A	N/A	0	N/A	N/A	N/A	N/A

*BMI, body mass index; F stage, fibrosis stage; HCC, hepatocellular carcinoma; mod, moderately differentiated; Mt, multiple tumor; NAFLD, non-alcoholic fatty liver disease; NAS, NAFLD activity score; N/A, not available; St, single tumor*.

The diagnosis of NAFLD was based on the presence of steatosis in the liver biopsy specimen (>5% of hepatocytes containing fat droplets) and exclusion of other causes of liver disease, such as viral hepatitis, alcoholic liver disease (quantity of ethanol intake >20 g/day for women and >30 g/day for men), drug-induced disease, or autoimmune liver disease (28). Patients were required to have no concomitant diseases or conditions that could cause secondary steatohepatitis, such as endocrine disorders, primary dyslipidemia, or malnutrition. Liver fibrosis stages (F0–4) were assessed according to Brunt's criteria (29). Aspartate aminotransferase to alanine transaminase ratio (30), aspartate aminotransferase to platelet ratio index score (31), fibrosis-4 score (32), gamma-glutamyl transpeptidase to platelet ratio (33, 34), and albumin-bilirubin grade (35) were calculated as previously reported.

### Isolation of Peripheral Blood and Intrahepatic Mononuclear Cells

Peripheral blood mononuclear cells (PBMCs) were isolated from whole blood samples using standard density gradient centrifugation on Ficoll-Paque (Nacalai Tesque, Kyoto, Japan). For isolation of intrahepatic lymphocytes (IHLs), non-cancerous liver tissues were enzymatically digested with DNase I (50 μg/L) (Promega, Madison, WI, USA) and collagenase IV (500 mg/L) (Nordmark Arzneimittel Gmbh & Co. KG, Uetersen, Germany) for 30–60 min at 37°C (36). PBMCs and IHLs were harvested and stored at −150°C in Cell Banker solution (ZENOAQ RESOURCE CO., LTD., Fukushima, Japan).

### Mass Cytometry (Cytometry Time-of-Flight, CyTOF)

**Figure 1:**
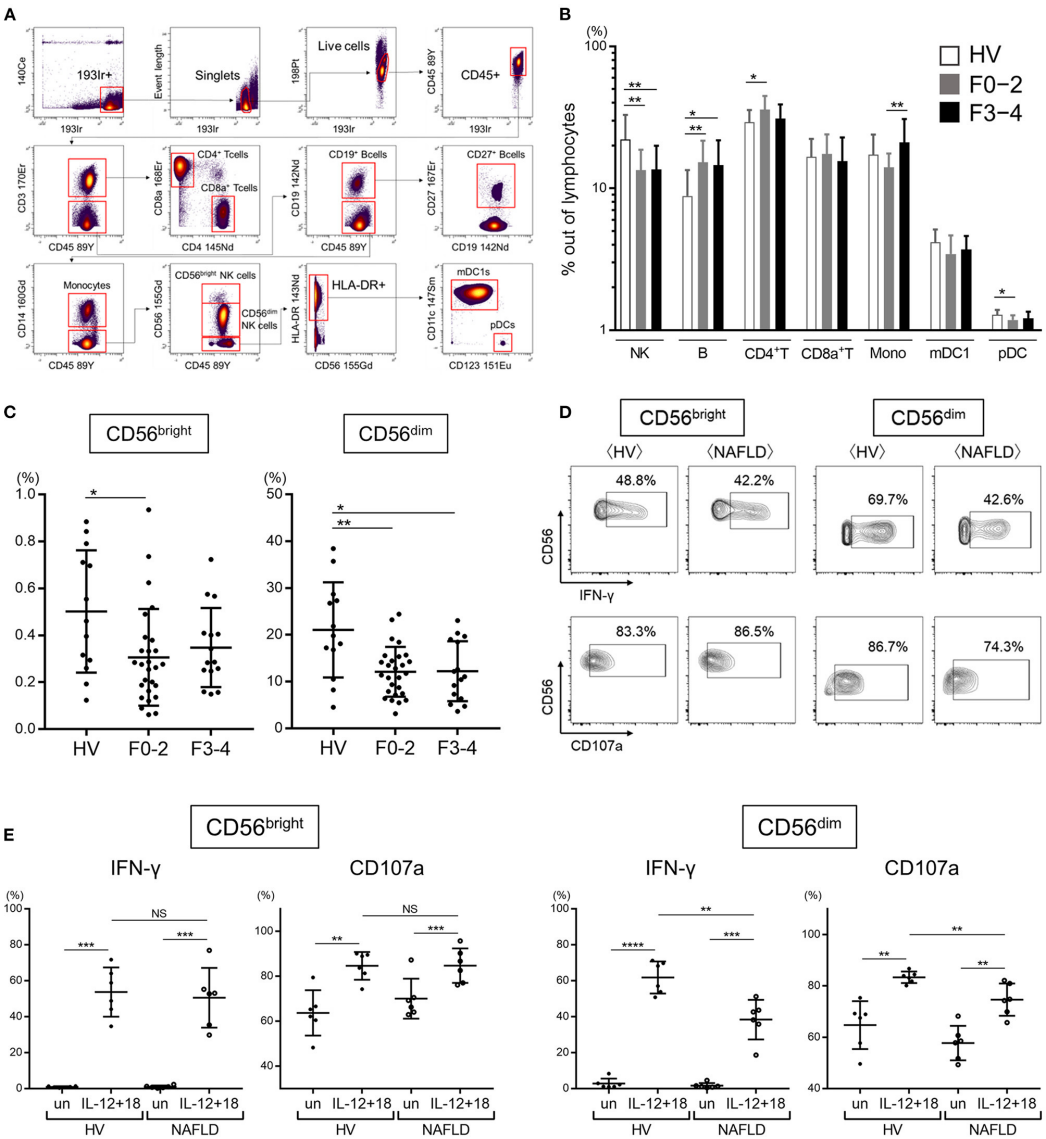
Reduced frequency and function of peripheral CD56^dim^ NK cells from NAFLD patients. **(A)** Mass cytometry gating scheme used to identify the peripheral blood immune cell subsets investigated here. Arrows indicate the gating sequence. **(B)** Summary of the frequency of the indicated immune cell populations isolated from healthy volunteers (HVs) (*n* = 13) and NAFLD patients with F0–2 (*n* = 27) or F3–4 (*n* = 15). **(C)** Percentage CD56^bright^ and CD56^dim^ NK cells among total peripheral blood mononuclear cells from HVs (*n* = 13) and NAFLD patients with F0–2 (*n* = 27) or F3–4 (*n* = 15). **(D)** Representative flow cytometry plots of CD56^bright^ and CD56^dim^ NK cells producing IFN-γ or expressing CD107a. **(E)** Quantification of IFN-γ-producing or CD107a-expressing CD56^bright^ and CD56^dim^ NK cells from NAFLD patients (*n* = 6) and HVs (*n* = 6) after incubation *in vitro* in the presence or absence (un) of IL-12 plus IL-18. Data in **(B,C,E)** are presented as the mean ± *SD*. *P < 0.05, **P < 0.01, ***P < 0.001 by the Mann–Whitney *U*-test. F0–2, fibrosis stage 0, 1, or 2; F3–4, fibrosis stage 3 or 4; HV, healthy volunteer; mDC1s, type 1 myeloid dendritic cells; Mono, monocytes; MSI, median signal intensity; NAFLD, non-alcoholic fatty liver disease; NK, natural killer; pDCs, plasmacytoid dendritic cells. ****P < 0.0001 by the Mann-Whitney *U*-test.

This procedure employs metal isotope-conjugated antibodies that can be distinguished by mass in a time-of-flight mass spectrometer, thereby allowing a large number of markers to be detected simultaneously without the spectral overlap limitations inherent to fluorophore-based flow cytometry. For analysis, the cells were thawed, incubated with cisplatin (Fluidigm, San Francisco, CA, USA) to identify live/dead cells, and then incubated with metal-conjugated antibodies to surface membrane proteins, as described below. The cells were fixed with 1.6% paraformaldehyde, labeled with an iridium-containing DNA intercalator to allow discrimination between singlets and doublets, and analyzed using a CyTOF mass cytometer (Fluidigm). CyTOF signals were normalized using EQ Beads (EQ Four Element Calibration Beads, 201078, Fluidigm) according to the manufacturer's instructions, and the data files were analyzed using Cytobank software (Mountain View, CA, USA). A total of 30,000 CD45^+^ leukocytes per sample was analyzed. A panel of 33 antibodies against the following markers were used for cell gating: immune cell subsets (CD3, CD4, CD8a, CD11c, CD14, CD19, CD56, CD123, and HLA-DR), Siglec family (Siglec-1,−2,−3,−5,−6,−7,−9,−10), NK cell differentiation markers (CD27, CD16, and CD57), NK cell activation markers (natural killer cell p30 (NKp30), natural killer cell p46 (NKp46), CD94/NK group 2 family of C-type lectin-like receptors (NKG2D), CD38, and CD69), and NK cell inhibitory markers [programmed cell death-1 (PD-1), immunoglobulin-like transcript 2 (ILT2), T-cell immunoreceptor with immunoglobulin and ITIM domains (TIGIT), T-cell immunoglobulin and mucin domain 3 (Tim-3), CD94/NK group 2 member A (NKG2A), killer immunoglobulin-like receptors (KIRs)] (Supplementary Table 1). Figure 1A shows the gating strategy used for identification of NK cells (CD45^+^, CD3^−^, CD19^−^, CD14^−^, and CD56^+^), CD4^+^ T cells (CD45^+^, CD3^+^, CD4^+^, and CD8a^−^), CD8^+^ T cells (CD45^+^, CD3^+^, CD4^−^, CD8a^+^), B cells (CD45^+^, CD3^−^, and CD19^+^), monocytes (CD45^+^, CD3^−^, CD19^−^, and CD14^+^), type 1 myeloid dendritic cells (mDC1s: CD45^+^, CD3^−^, CD19^−^, CD14^−^, CD56^−^, HLA-DR^+^, CD11c^+^, and CD123^−^), and plasmacytoid dendritic cells (pDCs: CD45^+^, CD3^−^, CD19^−^, CD14^−^, CD56^−^, HLA-DR^+^, CD11c^−^, and CD123^+^). Data files generated from the CyTOF analysis were subjected to a dimension reduction process based on the viSNE algorithm, allows multidimensional cytometry data to be presented in two dimensions while retaining the multidimensional data structure (37). For the viSNE analysis, 10,000 CD56^+^ NK cells from each donor were included and the data were clustered based on median signal intensity (MSI).

### *In vitro* Cytokine Production and Degranulation Assays

NK cell function was analyzed by flow cytometric staining of intracellular IFN-γ and cell surface CD107a, a lytic granule protein that is transported to the cell surface during degranulation. PBMCs from NAFLD patients and HVs were resuspended in RPMI 1640 medium (Thermo Fisher Scientific, Waltham, MA, USA) supplemented with 10% fetal bovine serum (GE Healthcare, Chicago, IL, USA), added to 96-well round-bottomed plates at 1.5 × 10^6^ cells/200 μL/well, and mixed with 1 μL/mL brefeldin A (BioLegend, San Diego, CA, USA), 20 ng/mL IL-12, and 20 ng/mL IL-18. The plates were incubated for 5 h at 37°C in a 5% CO_2_ incubator in the presence of APC-Cy7-conjugated anti-CD107a/LAMP-1 antibody (BioLegend). The cells were subsequently stained for 5 h at 37°C with antibodies against CD3-PerCP (BioLegend), CD56-V450 (BD, Franklin, NJ, USA), CD57-PE/Dazzle594 (BioLegend), PD-1-FITC (BioLegend), and Siglec-7-APC (BioLegend) for 30 min at 4°C; washed, fixed and permeabilized with Permeabilization Wash Buffer (BioLegend); and incubated for 30 min at 4°C with PE-conjugated anti-IFN-γ antibody (BioLegend) or isotype control antibody (mouse IgGκ1). Cells were analyzed on an LSRFortessa (BD Biosciences, San Jose, CA, USA) with FACSDiva software. Data were compensated using a compensation matrix generated with antibody-stained control beads. Final data analysis was performed using FlowJo 10.4.1 software (TreeStar, Ashland, OR, USA).

### Statistical Analysis

Differences between two groups were evaluated by the Mann–Whitney *U*-test or paired Student's *t*-test. Differences between more than two groups were evaluated by the Kruskal–Wallis test with Dunn's multiple comparison test. Correlations were assessed using Spearman's analysis. All analyses were performed using Prism software version 7 (Graph Pad, San Diego, CA, USA).

## Results

### Reduced Frequency and Function of Peripheral Blood NK Cells From Patients With NAFLD

We first performed CyTOF to determine the frequencies of the major immune cell subsets (NK cells, B cells, CD4^+^ T cells, CD8^+^ T cells, monocytes, mDC1s, and pDCs) in the peripheral blood of 13 HVs and 42 NAFLD patients, of whom 27 had mild or no fibrosis (F0–2) and 15 had advanced fibrosis (F3–4). An example of the gating strategy is shown in Figure 1A. Quantification of the cell frequencies indicated that total (CD56^+^) NK cells were significantly reduced in NAFLD patients compared with HVs, but there was no difference in total NK cell frequency between patients with early fibrosis (F0–2) and advanced fibrosis (F3–4) (Figure 1B). Compared with HVs, NAFLD patients exhibited significantly higher frequencies of B cells and CD4^+^ T cells, and a significantly lower frequency of pDCs, although the latter was observed only in patients with F0–2 (Figure 1B). Among the NK cell subsets, both CD56^bright^ and CD56^dim^ NK cells were decreased in patients with NAFLD, regardless of the fibrosis stage, compared with HVs (Figure 1C). The frequency of peripheral NK cells was lower in NAFLD patients with liver steatosis but was unaffected by the degree of liver inflammation or hepatocyte ballooning (Supplementary Figure 1).

Next, we evaluated the functional capacity of CD56^bright^ and CD56^dim^ NK cells by staining for intracellular IFN-γ or cell surface CD107a after *in vitro* stimulation with IL-12 and IL-18. Interestingly, IFN-γ production and CD107a expression were increased in both NK cell subpopulations from both HVs and NAFLD patients, but the increase was significantly more modest in the CD56^dim^ NK cell subset from NAFLD patients (Figures 1D,E). These findings revealed that while NAFLD was associated with a reduced frequency of both CD56^bright^ and CD56^dim^ NK cells, the function of only CD56^dim^ NK cells was compromised by NAFLD.

### Decreased Expression of Siglec-7 and Siglec-9 on Peripheral NK Cells From Patients With NAFLD

**Figure 2:**
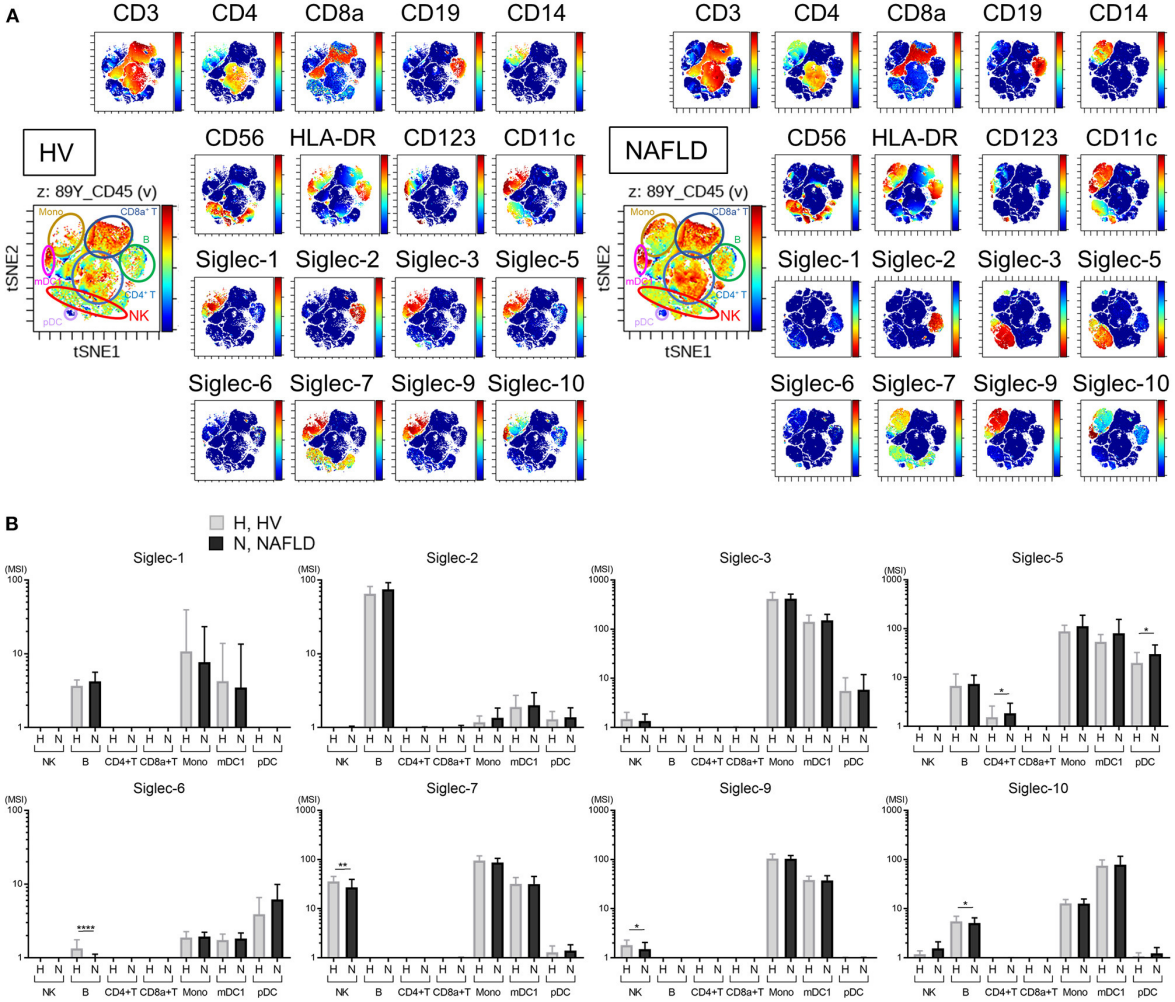
Decreased expression of Siglec-7 and Siglec-9 on peripheral blood NK cells from NAFLD patients. **(A)** Representative viSNE plots of total peripheral blood leukocytes (CD45^+^) from HVs. Left panel shows the identification of individual cell subpopulations after staining with a panel of cell type-specific markers. Right panels show expression of the indicated surface proteins on each cell subpopulation. The color scale indicates a gradient of high (red) to low (blue) expression level of the relevant protein. **(B)** Expression levels (MSI) of Siglecs on the indicated cell populations from HVs (*N* = 13) and NAFLD patients (*N* = 42). Data in **(B)** are presented as the means ± *SD*. *P < 0.05, **P < 0.01, ****P < 0.0001 by the Mann–Whitney *U*-test. B, B cells; HV, healthy volunteer; mDC1s, type 1 myeloid dendritic cells; Mono, monocytes; MSI, median signal intensity; NAFLD, non-alcoholic fatty liver disease; NK, natural killer cells; pDCs, plasmacytoid dendritic cells; Siglec, sialic acid-binding immunoglobulin-like lectin; tSNE, t-distributed stochastic neighbor embedding.

Representative viSNE plots of CyTOF analysis of PBMCs from three HVs and NAFLD patients are shown in Figure 2A. Total CD45^+^ PBMCs could be readily separated into seven cell subsets of NK cells, B cells, CD4^+^ T cells, CD8^+^ T cells, mDC1s, pDCs, and monocytes. Evaluation of Siglec-1, −2, −3, −5, −6, −7, −9, and −10 expression on each cell subset revealed detectable expression of Siglec-1 on B cells, monocytes, and mDC1s; Siglec-2 only on B cells; Siglec-3 on monocytes and mDC1s; Siglec-5 on monocytes, mDC1s, pDCs, and, at low levels, on B cells and CD4^+^ T cells; Siglec-6 on B cells, monocytes, mDC1s, and pDCs; Siglec-7 on NK cells, monocytes, and mDC1s; Siglec-9 on monocytes and mDC1s, and at a low level on NK cells; and Siglec-10 on B cells, monocytes, and mDC1s (Figure 2B). Of note, the expression levels of Siglec-7 and Siglec-9 were significantly lower on NK cells from NAFLD patients compared with HVs (Figure 2B). Additionally, NAFLD patients exhibited reduced Siglec-6 expression on B cells and elevated Siglec-5 expression on pDCs compared with HVs (Figure 2B).

### Decreased Expression of Siglec-7, Siglec-9, NKp30, and NKp46 and Increased Expression of CD57, PD-1, and ILT2 on Peripheral CD56^dim^ NK Cells From Patients With NAFLD

**Figure 3:**
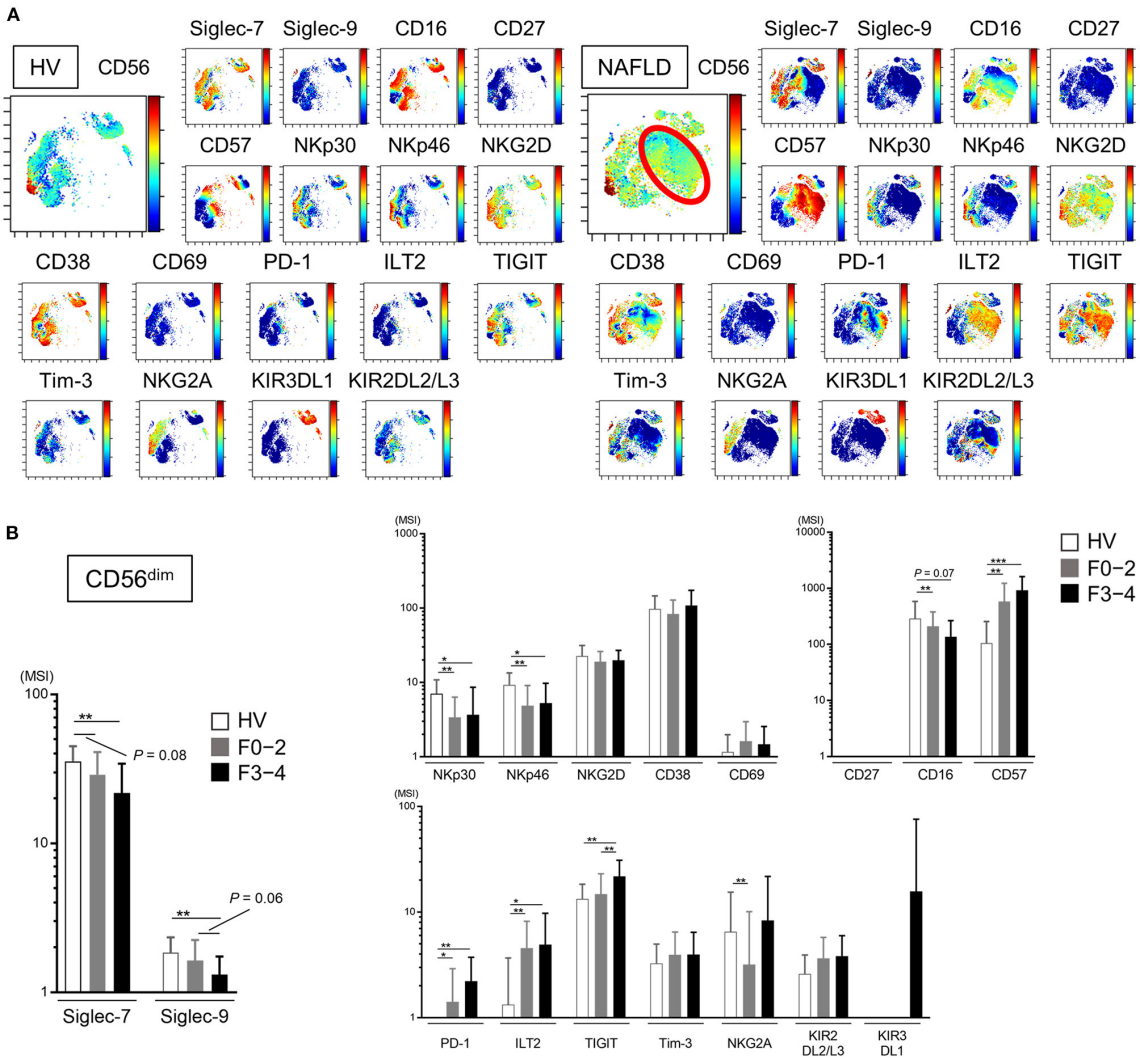
Phenotypic characterization of peripheral blood NK cells from NAFLD patients. **(A)** Representative viSNE plots of peripheral blood NK cells (CD56+) from HVs and NAFLD patients. Left panels indicate the distribution of CD56-expressing cells. The red circle indicates the CD56^dim^ NK subpopulation specifically detected in NAFLD patients. Right panels show expression of the indicated surface proteins among the NK cell subpopulations. The color scale indicates a gradient of high (red) to low (blue) expression level of the relevant protein. **(B)** Expression levels (MSI) of Siglecs and differentiation, activation, and inhibitory markers on CD56^dim^ NK cells from HVs (*n* = 13) and NAFLD patients with F0–2 (*n* = 27) or F3–4 (*n* = 15). Data are presented as the means ± *SD*. *P < 0.05, **P < 0.01, ***P < 0.001 by the Mann–Whitney *U*-test. F0–2, liver fibrosis stage 0–2; F3–4, liver fibrosis stage 3–4; HV, healthy volunteer; MSI, median signal intensity; NAFLD, non-alcoholic fatty liver disease; Siglec, sialic acid-binding immunoglobulin-like lectin; tSNE, t-distributed stochastic neighbor embedding.

We next analyzed the expression of a panel of NK cell differentiation, activation, and inhibition markers on cells from NAFLD patients and HVs. Using viSNE analysis of CyTOF data, we identified a CD56^dim^ NK subpopulation specifically present in NAFLD patients (Figure 3A, red circle). This CD56^dim^ subpopulation was characterized as Siglec-7^dim^Siglec-9^−^CD57^high^NKp30^−^NKp46^−^ILT2^+^TIGIT^+^Tim-3^−^and was notable for the fact that the inhibitory receptor PD-1 was mainly expressed on this NK cell subpopulation (Figure 3A). The expression levels (MSI) of Siglec-7 and Siglec-9 on CD56^dim^ NK cells were lower in NAFLD patients compared with HVs, and the reduction was more marked in patients with advanced fibrosis (Figure 3B). NKp30 and NKp46 expression levels were also reduced on CD56^dim^ NK cells in NAFLD patients, regardless of fibrosis stage, compared with HVs (Figure 3B). In contrast, some markers were upregulated on CD56^dim^ NK cells from NAFLD patients compared with HVs; among these, levels of CD57, PD-1, and ILT2 increased as fibrosis advanced (Figure 3B). Moreover, Siglec-7 expression on CD56^bright^ NK cells increased in patients with advanced fibrosis, which differed from the finding with CD56^dim^ cells (Supplementary Figure 2 and Figure 3B), whereas Siglec-9, NKp30, NKp46, PD-1, and ILT2 were expressed at comparable levels on CD56^bright^ NK cells from NAFLD patients and HVs (Supplementary Figure 2). TIGIT expression was elevated on CD56^dim^ and CD56^bright^ NK cells in patients with advanced fibrosis, but was comparable between cells from HVs and patients with little or no fibrosis (Figure 3B and Supplementary Figure 2). Finally, NKG2D, CD69, Tim-3, KIR3DL1, and KIR2DL2/L3 expression levels were comparable on CD56^dim^ NK cells from NAFLD patients and HVs (Figure 3B). Taken together, these data indicate that NAFLD is associated with downregulation of Siglec-7, Siglec-9, NKp30, and NKp46 and upregulation of CD57, PD-1, and ILT2 on CD56^dim^ NK cells (Figure 3B). Moreover, Siglec-7 levels on CD56^dim^ NK cells correlated positively with levels of the activation molecules Siglec-9, NKp30, and NKp46, and negatively with levels of the inhibitory molecules CD57, PD-1, and ILT2 (Supplementary Figure 3), suggesting that Siglec-7 could be a surrogate marker of the functional status of CD56^dim^ NK cells in NAFLD patients.

### Increased Frequency of Functionally Exhausted Peripheral Blood Siglec-7^–^CD57^+^PD-1^+^CD56^dim^ NK Cells From Patients With NAFLD

**Figure 4:**
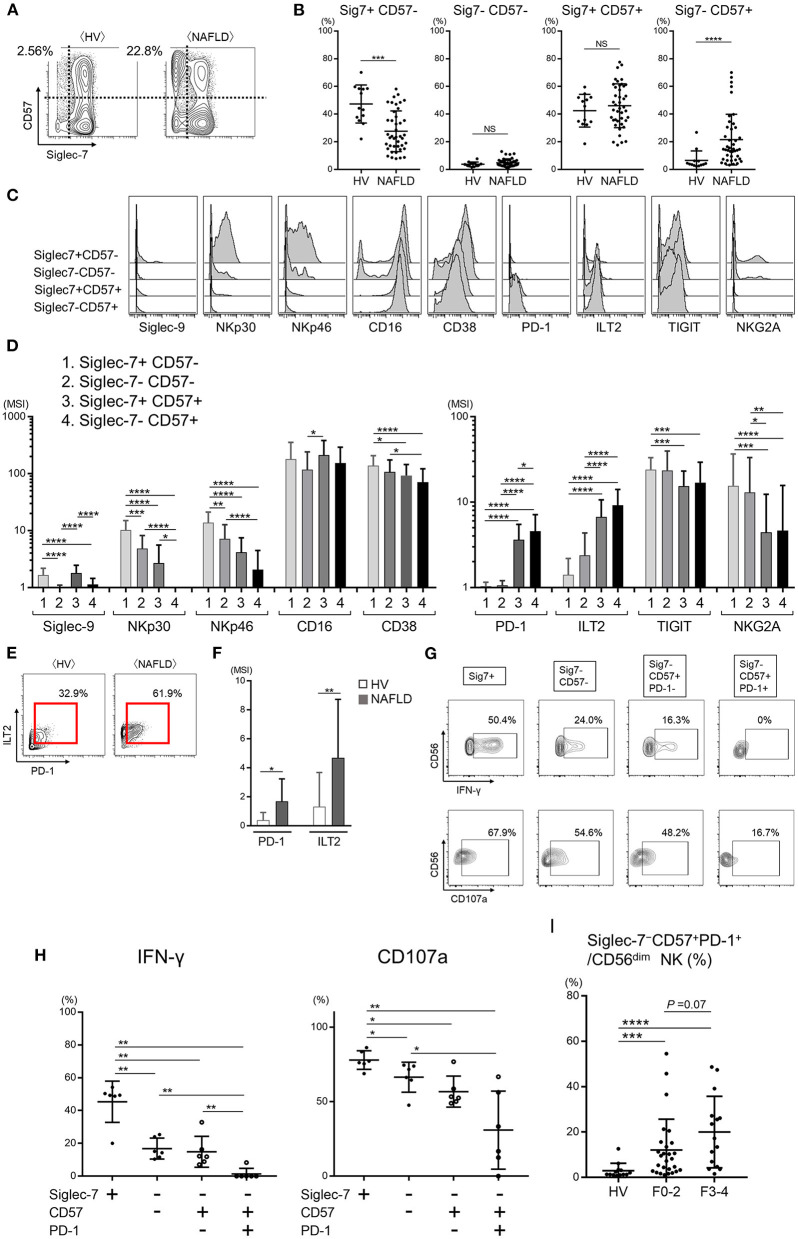
Dysfunction of the Siglec-7^−^CD57^+^PD-1^+^ subset of CD56^dim^ NK cells from NAFLD patients. **(A)** Representative mass cytometry plots of Siglec-7 and CD57 expression on CD56^dim^ NK cells from HVs and NAFLD patients. **(B)** Percentage Siglec-7^+^CD57^−^, Siglec-7^−^CD57^−^, Siglec-7^+^CD57^+^, and Siglec-7^−^CD57^+^ cells within the CD56^dim^ NK cell population from HVs (*n* = 13) and NAFLD patients (*n* = 42). **(C)** Representative histograms showing expression of the indicated cell surface proteins in the four CD56^dim^ NK cell subpopulations described in **(B)**. **(D)** Quantification of the data presented in **(C)** for the 42 NAFLD patients. **(E)** Representative mass cytometry plots showing PD-1 and ILT2 expression on the Siglec-7^−^CD57^+^ subset of CD56^dim^ NK cells. **(F)** Quantification of PD-1 and ILT2 expression (MSI) on Siglec-7^−^CD57^+^CD56^dim^ NK cells from HVs (*n* = 13) and NAFLD patients (*n* = 42). **(G)** Representative flow cytometry plots showing IFN-γ production and CD107a expression on the four CD56^dim^ NK cell subsets from NAFLD patients after stimulation *in vitro* with IL-12 and IL-18. **(H)** Quantification of the data shown in **(G)** for cells from 6 NAFLD patients. **(I)** Percentage Siglec-7^−^CD57^+^PD-1^+^CD56^dim^ NK cells from HVs (*n* = 13) and NAFLD patients according to fibrosis stage F0–2 (*n* = 27) or F3–4 (*n* = 15). Data are presented as the means ± SEM **(B,H,I)** or means ± *SD* **(B,D,F,H,I)**. *P < 0.05, **P < 0.01, ***P < 0.001, ****P < 0.0001 by the Mann–Whitney *U*-test **(B,F,H,I)** or the Kruskal–Wallis test with Dunn's multiple comparison test **(D)**. HV, healthy volunteer; ILT2, immunoglobulin-like transcript 2; MSI, median signal intensity; NAFLD, non-alcoholic fatty liver disease; NKG2A, CD94/NK group 2 member A; NS, not significant; PD-1, programmed cell death-1; Siglec, sialic acid-binding immunoglobulin-like lectin; Sig7, Siglec-7; TIGIT, T-cell immunoreceptor with Ig and ITIM domains.

As noted above, nearly all peripheral CD56^dim^ NK cells from HVs expressed Siglec-7, whereas NAFLD patients contained a significant subset of Siglec-7^−^CD56^dim^ NK cells (Figures 2C, 3B, and Supplementary Figure 4A). CD57, a marker of NK cell maturation, was strongly expressed on CD56^dim^ NK cells from NAFLD patients, particularly those with more advanced fibrosis (Figures 3A,B). Therefore, we next analyzed the frequency of CD56^dim^ NK cell subpopulations classified as positive or negative for Siglec-7 and CD57 expression. While NAFLD patients and HVs both contained approximately the same frequencies of Siglec-7^−^CD57^−^ and Siglec-7^+^CD57^+^ CD56^dim^ NK cell subsets, the Siglec-7^+^CD57^−^CD56^dim^ subset was significantly decreased and the Siglec-7^−^CD57^+^ subset was significantly increased in NAFLD patients compared with HVs (Figures 4A,B). An increase in Siglec-7^−^CD57^+^ cells was also observed among the CD56^bright^ NK cell subpopulation in NAFLD patients, although to a more modest degree than that seen with CD56^dim^ NK cells (Supplementary Figures 4B–D). Within the peripheral CD56^dim^ NK cell population from NAFLD patients, the Siglec7^−^CD57^+^ and Siglec7^+^CD57^+^ subsets expressed much lower levels of NKp30 and NKp46 and higher levels of PD-1 and ILT2 compared with the Siglec7^+^CD57^−^ and Siglec7^−^CD57^−^ subsets (Figures 4C,D). Moreover, PD-1 and ILT2 levels were higher on Siglec7^−^CD57^+^CD56^dim^ NK cells from NAFLD patients compared with HVs (Figures 4E,F). With respect to NK cell function, IFN-γ and CD107a expressions were impaired in the Siglec7^−^CD56^dim^ subsets (Siglec-7^−^CD57^−^, Siglec-7^−^CD57^+^PD-1^−^, and Siglec-7^−^CD57^+^PD-1^+^ CD56^dim^) compared with the Siglec7^+^CD56^dim^ subset from NAFLD patients (Figures 4G,H), as previously reported (26), and the Siglec-7^−^CD57^+^PD-1^+^ subset were functionally impaired compared with either Siglec7^−^CD57^−^ or Siglec7^−^CD57^−^PD-1^−^ subsets of CD56^dim^ cells (Figures 4G,H). IFN-γ expressions in each NK subset were more impaired in NAFLD patients than those in HVs (Supplementary Figure 4E). Finally, the frequency of Siglec-7^−^CD57^+^PD-1^+^CD56^dim^ cells was significantly higher in NAFLD patients than in HVs, even among patients with mild or no fibrosis (Figure 4I). Taken together, these results indicated not only that peripheral blood Siglec-7^−^CD57^+^PD-1^+^CD56^dim^ NK cells were highly dysfunctional but also that the frequency of this NK cell subset was increased in NAFLD patients compared with HVs.

### Increased Expression of NKG2D and CD69 on Intrahepatic NK Cells in Patients With NAFLD

**Figure 5:**
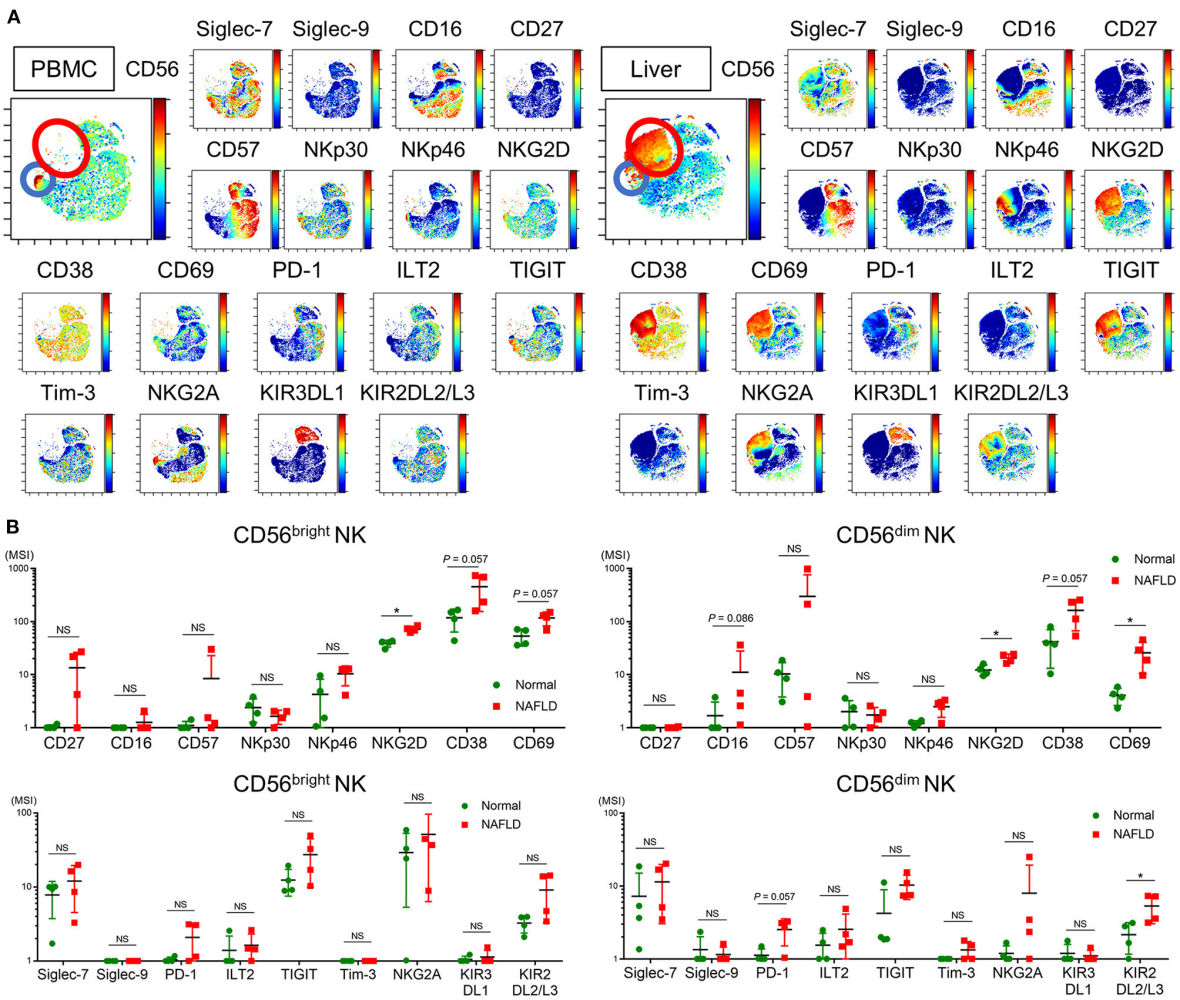
Phenotype of intrahepatic and peripheral blood NK cells from NAFLD patients. **(A)** Representative ViSNE plots of total CD56^+^ NK cells in the peripheral blood and liver from NAFLD patients. Left panels indicate the distribution of CD56-expressing cells. Right panels show expression of the indicated surface proteins on each NK cell subpopulation. The color scale indicates a gradient of high (red) to low (blue) expression level of the relevant protein. The red circle indicates the CD56^bright^ subpopulation specifically detected in the liver of NAFLD patients. The color scale indicates a gradient of high (red) to low (blue) expression of the relevant protein. **(B)** Expression levels (MSI) of 17 surface markers on CD56^bright^ and CD56^dim^ NK cell subpopulations isolated from NAFLD patient-derived (*n* = 4) and normal (*n* = 4) livers. Data are presented as the means ± *SD*, with individual subjects represented as squares. *P < 0.05 by the Mann–Whitney *U*-test. ILT2, immunoglobulin-like transcript 2; KIR2DL2/L3, killer cell immunoglobulin-like receptor 2DL2/L3; KIR3DL1, killer cell immunoglobulin-like receptor, three Ig domains and long cytoplasmic tail 1; MSI, median signal intensity; NAFLD, non-alcoholic fatty liver disease; NKG2A, CD94/NK group 2 member A; NKG2D, CD94/NK group 2 family of C-type lectin-like receptors; NS, not significant; PBMC, peripheral blood mononuclear cell; PD-1, programmed cell death-1; Siglec, sialic acid-binding immunoglobulin-like lectin; TIGIT, T-cell immunoreceptor with Ig and ITIM domains; Tim-3, T-cell immunoglobulin and mucin domain 3.

NK cells are enriched in the liver, and the cell surface phenotype of intrahepatic NK cells is reported to differ from that of peripheral NK cells (38). Because our investigation thus far has focused on peripheral blood NK cells, we next compared the frequency and phenotype of NK cells from peripheral blood and non-cancerous liver samples from patients who underwent liver resection for NAFLD-associated HCC (Table 2). viSNE analysis of CyTOF data identified a CD56^bright^ NK cell population that was much more abundant in the liver compared with peripheral blood (Figure 5A, red and blue circles, respectively) and differed markedly in surface marker expression. Intrahepatic CD56^bright^ NK cells showed increased expression of CD69 and TIGIT and reduced expression of NKp30 and NKG2A compared with peripheral CD56^bright^ NK cells (Supplementary Figure 5). Moreover, Siglec-7 expression was lower in CD56^bright^ and CD56^dim^ NK cell subpopulations present in the liver compared with the equivalent peripheral NK cell subpopulations, although the differences did not reach the level of statistical significance (Supplementary Figure 5).

We next compared the phenotypes of NK cells from the livers of NAFLD patients and donors undergoing resection of metastatic liver tumors unrelated to NAFLD (normal liver). The frequency of intrahepatic Siglec-7^−^CD57^+^PD-1^+^CD56^dim^ NK cells was comparable between NAFLD and normal liver specimens (data not shown), which contrasted with the findings for the same subpopulations in peripheral blood (Figure 4I). However, intrahepatic CD56^dim^ NK cells from NAFLD patients displayed significantly increased levels of CD16, NKG2D, CD38, CD69, PD-1, ILT2, TIGIT, and KIR3DL1 compared with normal intrahepatic NK cells (Figure 5B). Increased CD69 expression on intrahepatic CD56^dim^ NK cells has also been reported in patients infected with HCV (39), suggesting that activated intrahepatic effector NK cells may contribute to liver inflammation and disease progression. The expression level of NKG2D on intrahepatic CD56^bright^ NK cells was also significantly higher in liver from NAFLD patients compared with normal liver (Supplementary Figure 5), as reported for HBV- and HCV-infected patients (40).

## Discussion

The goal of this study was to clarify the phenotype–function relationship of NK cells from patients with NAFLD, with a particular focus on immune-modulating proteins, including Siglecs. We showed that NAFLD was associated with decreased frequencies of total CD56^+^, CD56^dim^, and CD56^bright^ NK cells and an increased frequency of Siglec-7^−^CD57^+^PD-1^+^CD56^dim^ NK cells. We also found that the peripheral CD56^dim^ and Siglec-7^−^CD57^+^PD-1^+^CD56^dim^ NK cell populations were both functionally impaired in NAFLD patients compared with HVs. Of note, many of the changes in NK cell subsets were evident even in patients with mild fibrosis.

The mechanisms underlying the decreased frequency of NK cells in NAFLD patients are unclear. The mean body mass index (BMI), age, and sex need to be considered as contributing factors. BMI and age were higher in our patient cohort compared with the HVs. Obesity is an acknowledged risk factor for progression from NAFLD to non-alcoholic steatohepatitis (NASH), development of fibrosis, and death (41). However, the impact of obesity on NK cell frequency is controversial. Two reports have demonstrated that the frequency of NK cells is reduced in morbidly obese adults (BMI >40 kg/m^2^) (42, 43), whereas other reports found no differences between obese and lean adults (BMI >30 and <25 kg/m^2^) groups (44, 45). In the present study, we detected no significant associations between peripheral CD56^+^ NK cell frequency and either BMI (data not shown) or fibrosis stage (Figure 1C). With regard to age, a significant positive association between age and both percentage and absolute number of CD3^−^CD56^+^ NK cells has been reported (46–49). Our NAFLD patients also showed a significant positive correlation between age and CD56^dim^NKcells and NKG2D expression on CD56^dim^NKcells (Supplementary Figure 6). As to sex, Phan MT et al. has reported that men showed greater NK proportion and numbers, frequencies of CD56^dim^ cells, and CD57 expression compared with those of women in a healthy Korean population (50). However, the frequency, function, and phenotype of CD56^dim^ NK cells showed differences between NAFLD patients and HVs, regardless of sex (data not shown). The frequency and function of NK cells are reduced in patients with chronic viral hepatitis (39), suggesting that persistent infection or liver inflammation could result in the exhaustion of NK cells. Stiglund et al. reported comparable frequencies of NK cells among HVs, patients with non-alcoholic fatty liver (median F0), and patients with NASH (median F2) of the same age and BMI (51). In contrast, Diedrich et al. reported an increased NK cell frequency in NAFLD patients compared with HVs (mean ages 51 and 38 years, respectively; fibrosis stage and BMI not reported) (52). However, it remains to be clarified whether such discordant findings with respect to the effects of obesity, age, and disease settings on NK cell frequency might be due to differences in subject demographics such as BMI, disease duration, and fibrosis stage.

We found reduced expression of Siglec-7 on CD56^dim^ NK cells from patients with NAFLD compared with HVs. Siglec-7 expression levels CD56^dim^ NK cells on showed no correlation with age, BMI, ALT, F stage, and NAS (Supplementary Figure 7). Of note is the finding that Siglec-7 expression level on this cell subset correlated positively with expression of exhaustion and inhibitory molecules (CD57, PD-1, and ILT2) and inversely with expression activation receptors (NKp30 and NKp46). Among these, CD57 is regarded as a marker of terminal NK differentiation (53). CD57 has been reported to be mainly expressed on CD4^+^ T cells, CD8^+^ T cells, and CD56^dim^ NK cells and to be elevated in subjects with advancing age, cancer, autoimmune diseases, or chronic hepatitis (54, 55). In patients with NASH, CD57^+^CD3^+^ NKT cells are reported to be increased in the liver (56). CD28^−^CD57^+^CD8_+_ T cells are increased in the development of diabetes and contribute to abnormal glucose homeostasis (57). However, the functional relevance of altered CD57 expression in these settings is unclear. We examined four subgroups of CD56^dim^ NK cells stratified by Siglec-7 and CD57 expression, and found that the frequency of Siglec-7^−^CD57^+^CD56^dim^ NK cells was higher in NAFLD patients compared with HVs. These data show that NK cells are phenotypically altered in NAFLD patientsdue to complicated multiple factors.

NK cells from patients with chronic HBV or HCV infection exhibit impaired cytokine production and cytolytic activity (40). In the present study, we found similar functional impairments among the CD56^dim^ NK cell population from NAFLD patients compared with HVs, and within the Siglec-7^−^ compared with Siglec-7^+^ subpopulation of CD56^dim^ NK cells from NAFLD patients. PD-1 is an immune checkpoint molecule that transmits inhibitory signals to both T cells and NK cells upon ligand engagement (58, 59). In our study, co-expression of PD-1 on Siglec7^−^CD57^+^CD56^dim^ NK cells was an indicator of further functional impairment. These results suggest that Siglec-7 and PD-1 are function-related markers for CD56^dim^ NK cells in patients with NAFLD. Indeed, the frequency of the dysfunctional Siglec-7^−^CD57^+^PD-1^+^CD56^dim^population was significantly increased in NAFLD patients even at the earliest stages of fibrosis (F0–2), but was unaffected by age, BMI, or metabolic complications such as hypertension, diabetes mellitus, and dyslipidemia (data not shown). Thus, the mechanisms underlying the NAFLD-associated increase in dysfunctional Siglec-7^−^CD57^+^PD-1^+^CD56^dim^ NK cells remain to be addressed.

The liver plays a crucial role in innate immunity, and, under normal conditions, it harbors a number of innate immune cells, including NK cells. The proportion and phenotype of intrahepatic immune cells are altered in patients with acute or chronic liver disease, such as viral hepatitis or steatohepatitis. In HCV- and HBV-infected individuals, intrahepatic NK cells display enhanced expression of NKG2D and CD69, and the levels correlate positively with serum alanine aminotransferase and HCV RNA levels (38). It is thus plausible that changes in intrahepatic NK cell phenotype and function could be associated with the pathogenesis of NAFLD. We showed that NAFLD was accompanied by increased frequencies of intrahepatic NKG2D^+^ and CD69^+^ NK cells, suggesting that hepatic NK cells are activated in this disease. However, the small sample number (*n* = 4) of our intrahepatic NK cell analysis limited our ability to investigate correlations between intrahepatic NK cell phenotypes or functions and clinical parameters. There are many reports on the distinct phenotype and function of NK cells between in the liver and in the periphery (38, 60, 61). In general, such spatial difference of NK cells may be augmented in patients with inflammatory liver disease, such as NAFLD. One of the key factors impacting on NK cells is IL-15. IL-15 is present constitutively in the hepatic microenvironment, and upregulated in viral hepatitis (62). IL-15 is mainly produced by Kupffer cells and monocytes/bone marrow-derived macrophages (62). IL-15 can restore the downregulated NK cell function in chronic diseases (63) and increases NKG2D expression on NK cells (64). Although we have not measured intra-hepatic expression of IL-15 in NAFLD patients, the previous reports support the possibility that up-regulated IL-15 might result in higher expression of NKG2D in the liver of patients with NAFLD.

Natalie Stiglund et al. showed the upregulation of NKG2D on peripheral NK cells from NASH patients as compared to NAFL patients (51). We compared NKG2D expression levels on peripheral NK cells between NASH and NAFL patients. The diagnosis of NASH was determined by the classification of Matteoni. As a result, the expression of NKG2D on NK cells from NASH patients was not different from those in NAFL patients and healthy volunteers (Supplementary Figure 8). One of the plausible reasons causing such discrepancy between two studies may be the difference of the F stage in the population of patient cohorts. Our study cohort enrolled more patients with advanced fibrosis compared to the study by Stiglund et al. The variation of NAFL and NASH patients in our cohort showed advanced fibrosis stage as compared to the cohort in the paper by them (Supplementary Table 2). In the NAFLD patients (NAFL/NASH) in our cohort, NK cells might be low-reactive in response to inflammatory stimuli due to long-lasting chronic inflammation, possibly resulting in lesser expression of NK activation marker NKG2D.

In summary, we have performed a comprehensive analysis of differences between the surface markers on NK cells in NAFLD patients and HVs and the relevance of such changes to cell function. We showed that the abundance and function of peripheral NK cells are decreased in patients with NAFLD, even those with mild fibrosis. We also showed that Siglec-7 and PD-1 are markers of CD56^dim^ NK cell function in NAFLD patients and that NAFLD is associated with an elevated frequency of NK cells with the dysfunctional Siglec-7^−^CD57^+^PD-1^+^CD56^dim^ phenotype. These findings merit further studies to investigate the relationship between impaired NK cell function and disease progression, with a possible goal of exploring the therapeutic utility of restoring CD56^dim^ NK cell function for NAFLD.

## Data Availability Statement

The raw data supporting the conclusions of this article will be made available by the authors, without undue reservation.

## Ethics Statement

The studies involving human participants were reviewed and approved by National Center for Global Health and Medicine under Clinical Research Number 2355, Nippon Medical School under Clinical Research Number 685, Nagoya University under Clinical Research Number 2019-0030-15255, Japanese Foundation for Cancer Research under Clinical Research Number 2017-1118. The patients/participants provided their written informed consent to participate in this study.

## Author's Note

The details of the observational studies can be found here: National Center for Global Health and Medicine under Clinical Research Number 2355 https://ccs.ncgm.go.jp/120/040/pdf/202005_rinri.pdf; Nippon Medical School under Clinical Research Number 685 https://www.nms.ac.jp/hokuso-h/info/safety/ethics-committee.html; Nagoya University under Clinical Research Number 2019-0030-15255 https://www.med.nagoya-u.ac.jp/medical_J/ethics/cat3637/rinsyoukansatsu.html; Japanese Foundation for Cancer Research under Clinical Research Number 2017-1118 https://www.jfcr.or.jp/up_pdf/20190116100654_1.pdf.

## Author Contributions

YS, SYoshio, HD, and TK: conception and design of the study. YS, YA, YY, TA, NI, MA, TI, TH, and YOs: acquisition of data. YS, SYoshio, HD, HK, TS, YY, YT, SYoshik, TM, MM, and YOs: analysis and interpretation of data. YS and SYoshio: drafting of the manuscript and statistical analysis. HD, HK, TS, YY, YT, SYoshik, TM, MM, and YOs: critical revision of the manuscript for important intellectual content. SYoshio, HD, YM, YOn, YT, AS, and AT: technical or material support. SYoshio and TK: study supervision. All authors contributed to the article and approved the submitted version.

## Conflict of Interest

TK received lecture fees from Gilead Sciences and Merck Sharp & Dohme. The remaining authors declare that the research was conducted in the absence of any commercial or financial relationships that could be construed as a potential conflict of interest.

## Glossary

BMI, body mass index; F stage, fibrosis stage; FcγRIIIa, Fc-gamma receptor IIIa; FIB-4, fibrosis-4; HBs, hepatitis B surface; HBV, hepatitis B virus; HCC, hepatocellular carcinoma; HCV, hepatitis C virus; HIV, human immunodeficiency virus; HV, healthy volunteer; IFN-γ, interferon-gamma; IHL, intrahepatic lymphocyte; IL, interleukin; ILT2, immunoglobulin-like transcript 2; KIR2DL2/L3, killer cell immunoglobulin-like receptor 2DL2/L3; KIR3DL1, killer cell immunoglobulin-like receptor, three Ig domains and long cytoplasmic tail 1; KIRs, killer immunoglobulin-like receptors; LAMP-1, lysosomal-associated membrane protein 1; mDC1, type 1 myeloid dendritic cell; MSI, median signal intensity; NAFL, non-alcoholic fatty liver; NAFLD, non-alcoholic fatty liver disease; NASH, non-alcoholic steatohepatitis; NK, natural killer; NKG2A, CD94/NK group 2 member A; NKG2D, CD94/NK group 2 family of C-type lectin-like receptors; NKp30, natural killer cell p30; NKp46, natural killer cell p46; PBMC, peripheral blood mononuclear cell; PD-1, programmed cell death-1; pDC, plasmacytoid dendritic cell; Siglec, sialic acid-binding immunoglobulin-like lectin; TIGIT, T-cell immunoreceptor with immunoglobulin and ITIM domains; Tim-3, T-cell immunoglobulin and mucin domain 3; t-SNE, t-distributed stochastic neighbor embedding; γGTP, gamma-glutamyl transpeptidase.
