# Supplementary material for: Increased Frequency of Dysfunctional Siglec-7−CD57+PD-1+ Natural Killer Cells in Patients With Non-alcoholic Fatty Liver Disease
Source: Front Immunol. 2021 Feb 22;12:603133. doi: 10.3389/fimmu.2021.603133 (PMC7938755; doi:10.3389/fimmu.2021.603133)
Supplement: Supplementary file 1 [file Data_Sheet_1.docx]

Supplementary Material

# Supplementary Table

## Supplementary Table 1. Antibodies used for mass cytometry

| Channel | Isotype | Marker | Function of Markers | Clone | Staining  Method | PC/IH |
| --- | --- | --- | --- | --- | --- | --- |
| 89 | Y | CD45 | lymphocytes identification | HI30 | surface | PC |
| 141 | Pr | Siglec-2 (CD22) | Siglec family | HIB22 | surface | IH |
| 142 | Nd | CD19 | naïve B identification | HIB19 | surface | PC |
| 143 | Nd | HLA-DR | DC identification | L243 | surface | PC |
| 144 | Nd | CD69 | NK activation | FN50 | surface | PC |
| 145 | Nd | CD4 | CD4 T identification | RPA-T4 | surface | PC |
| 147 | Sm | CD11c | mDC identification | Bu15 | surface | PC |
| 148 | Nd | Siglec-10 | Siglec family | Not described | surface | IH |
| 149 | Sm | Siglec-9 (CD329) | Siglec family | K8 | surface | IH |
| 150 | Nd | Siglec-6 (CD327) | Siglec family | 767329 | surface | IH |
| 151 | Eu | CD123 (IL-3R) | pDC identification | 6H6 | surface | PC |
| 152 | Sm | Siglec-7 (CD328) | Siglec family | 194211 | surface | PC |
| 153 | Eu | TIM-3 | NK inhibitory | F38-2E2 | surface | PC |
| 154 | Sm | TIGIT | NK inhibitory | MBSA43 | surface | PC |
| 155 | Gd | CD56 (NCAM) | NK identification | B159 | surface | PC |
| 156 | Gd | ILT2 (CD85j) | NK inhibitory | GHI/75 | surface | PC |
| 158 | Gd | Siglec-1 (CD169) | Siglec family | 7-239 | surface | PC |
| 159 | Tb | NKp30 (CD337) | NK activation | Z25 | surface | PC |
| 160 | Gd | CD14 | monocytes identification | M5E2 | surface | PC |
| 161 | Dy | Siglec-5 (CD170) | Siglec family | 194128 | surface | IH |
| 162 | Dy | NKp46 (CD335) | NK activation | BAB281 | surface | PC |
| 163 | Dy | Siglec-3 (CD33) | Siglec family | WM53 | surface | PC |
| 164 | Dy | KIR3DL1 (CD158e1) | NK inhibitory | DX9 | surface | IH |
| 166 | Er | NKG2D (CD314) | NK activation | ON72 | surface | PC |
| 167 | Er | CD27 | memory B identification | O323 | surface | PC |
| 168 | Er | CD8a | CD8a T identification | SK1 | surface | PC |
| 169 | Tm | NKG2A (CD159a) | NK inhibitory | Z199 | surface | PC |
| 170 | Er | CD3 | T identification | UCHT1 | surface | PC |
| 172 | Yb | CD38 | plasma B identification | HIT2 | surface | PC |
| 173 | Yb | KIR2DL2/L3 (CD158b) | NK inhibitory | DX27 | surface | PC |
| 174 | Yb | PD-1 (CD279) | NK inhibitory | EH12.2H7 | surface | PC |
| 176 | Yb | CD57 | NK inhibitory | HCD57 | surface | PC |
| 209 | Bi | CD16 | cytotoxicity NK identification | 3G8 | surface | PC |

HLA-DR, human leukocyte antigen-DR; IH, in-house (labeling kit); ILT2, immunoglobulin-like transcript 2; KIR2DL2/L3, killer cell immunoglobulin-like receptor 2DL2/L3; KIR3DL1, killer cell immunoglobulin-like receptor, three Ig domains and long cytoplasmic tail 1; NKG2A, CD94/NK group 2 member A; NKG2D, CD94/NK group 2 family of C-type lectin-like receptors; PC, preconjugated; PD-1, programmed cell death-1; Siglec, sialic acid-binding immunoglobulin-like lectin; TIGIT, T-cell immunoreceptor with Ig and ITIM domains; Tim-3, T-cell immunoglobulin and mucin domain 3.

## Supplementary Table 2. NAFL/NASH patient characteristics

|  | **NAFL^a^** | **NASH^b^** |
| --- | --- | --- |
| Subjects | 13 | 24 |
| Age (year), mean | 64 | 58.5 |
| Male | 7 (53.8%) | 6 (25.0%) |
| BMI (kg/m^2^), mean | 29.9 | 28.6 |
| ALT (IU/L), median | 46 | 62.5 |
| NAFLD Activity Score, median (range) | 2 (1-4) | 4 (2-6) |
| Steatosis (0-3), median (range) | 1 (0-2) | 1 (0-2) |
| Lobular inflammation (0-3), median (range) | 1 (0-2) | 2 (1-3) |
| Hepatocyte ballooning (0-2), median (range) | 0 (0) | 1 (1-2) |
| Fibrosis score (0-4)^c^ | F0 (N=2) F1 (N=7) F2 (N=2) F3 (N=2) | F0 (N=1) F1 (N=4) F2 (N=11) F3 (N=5) F4 (N=3) |
| ^a^Non-alcoholic fatty liver. |  |  |
| ^b^Non-alcoholic steatohepatitis. |  |  |
| ^c^Fibrosis score according to Brunt's criteria (29) |  |  |

Data are presented as patient number, score (range) as appropriate. ALT, alanine aminotransferase; BMI, body mass index; NAFL, non-alcoholic fatty liver; NAFLD, non-alcoholic fatty liver disease; NASH, non-alcoholic steatohepatitis.

# Supplementary Figures

## Supplementary Figure 1

**
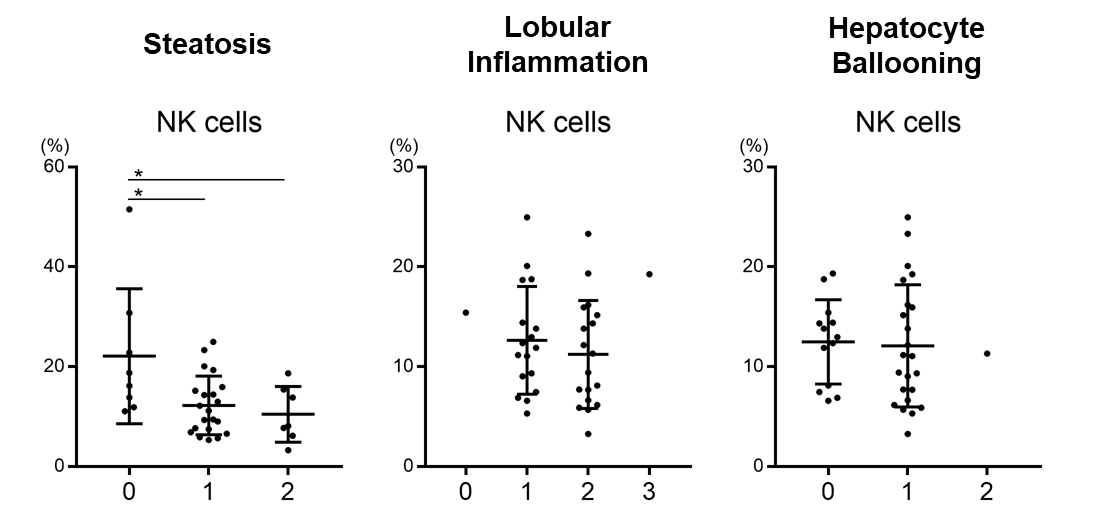
**

**Figure S1. Frequency of peripheral CD56^+^ NK cells from NAFLD patients stratified by severity of hepatic steatosis, lobular inflammation, and ballooning**

Flow cytometric analysis of CD56^+^ NK cells from NAFLD patients (n=42) with the indicated scores for each condition. Data are presented as the means ± SD with individual patients represented as circles. **P* < 0.05 by the Mann–Whitney U-test.

## Supplementary Figure 2

**
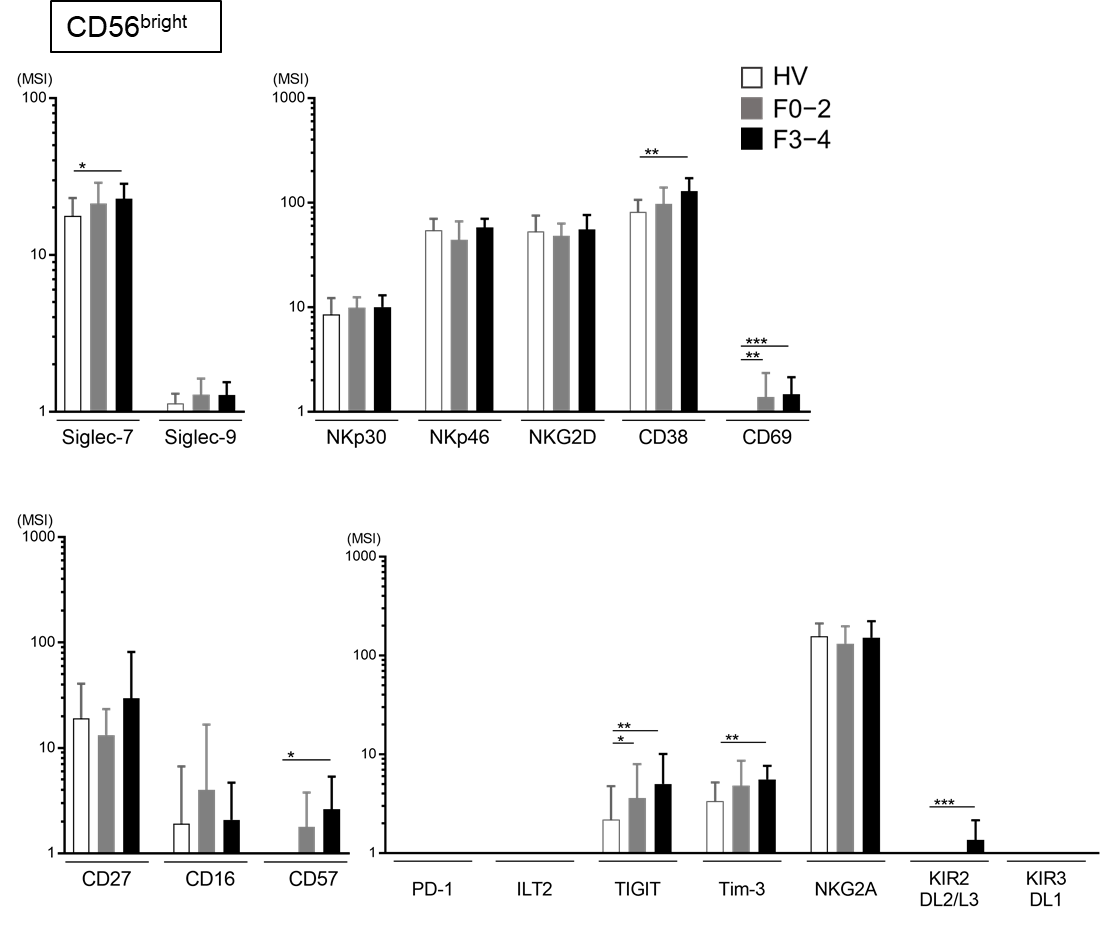
**

**Figure S2. Expression of surface markers on peripheral CD56^bright^ NK cells**

Expression levels (MSI) of the indicated Siglecs and differentiation, activation, and inhibitory markers on CD56^bright^ NK cells from HVs (n=13) and NAFLD patients with F0–2 (n=27) or F3–4 (n=15). Data are presented as the means ± SD. **P* < 0.05, ***P* < 0.01, ****P* < 0.001 by the Mann–Whitney U-test. F0–2, liver fibrosis stage 0–2; F3–4, liver fibrosis stage 3–4; HV, healthy volunteer; MSI, median signal intensity; NAFLD, non-alcoholic fatty liver disease; Siglec, sialic acid-binding immunoglobulin-like lectin.

## Supplementary Figure 3

**
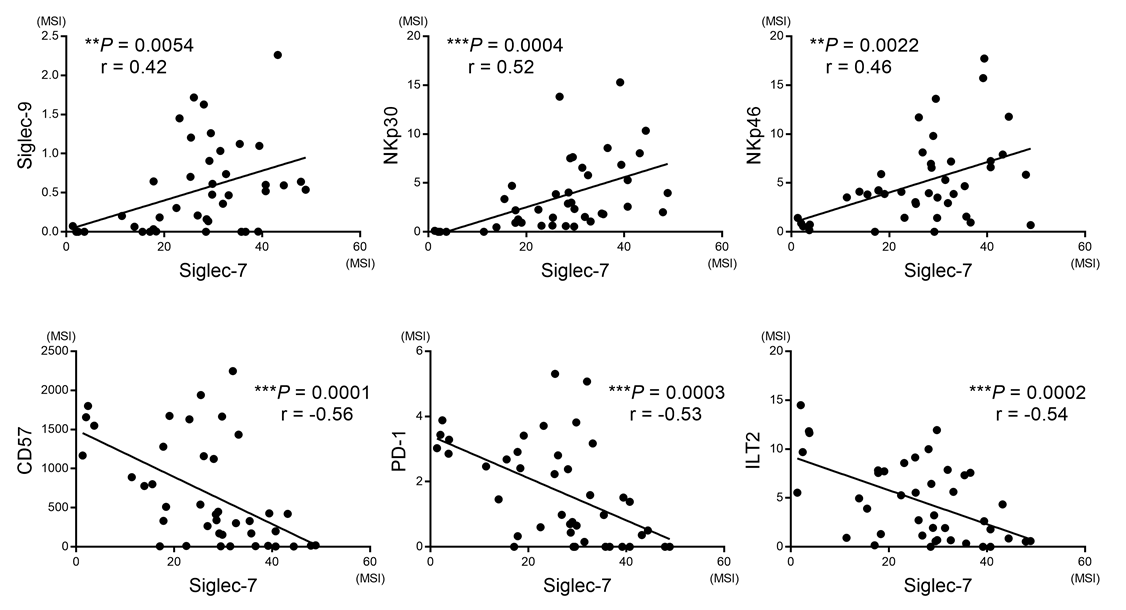
**

**Figure S3. Correlations between expression of Siglec-7 and Siglec-9, NKp30, NKp46, CD57, PD-1, and ILT2 in CD56^dim^ NK cells from NAFLD patients**

Expression levels (MSI) were evaluated for 42 NAFLD patients. *P* values and correlation coefficients (r) were calculated with Spearman’s correlation test. ILT2, immunoglobulin-like transcript 2; MSI, median signal intensity; NAFLD, non-alcoholic fatty liver disease; PD-1, programmed cell death-1; Siglec, sialic acid-binding immunoglobulin-like lectin.

## Supplementary Figure 4

**
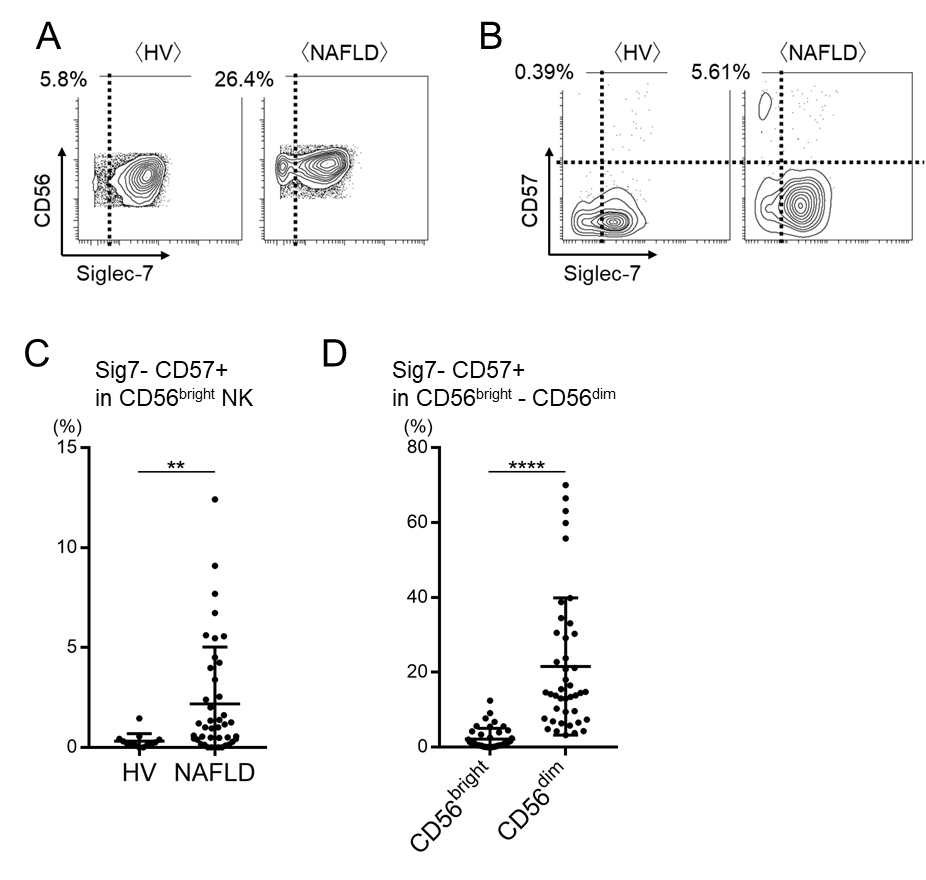
**

**
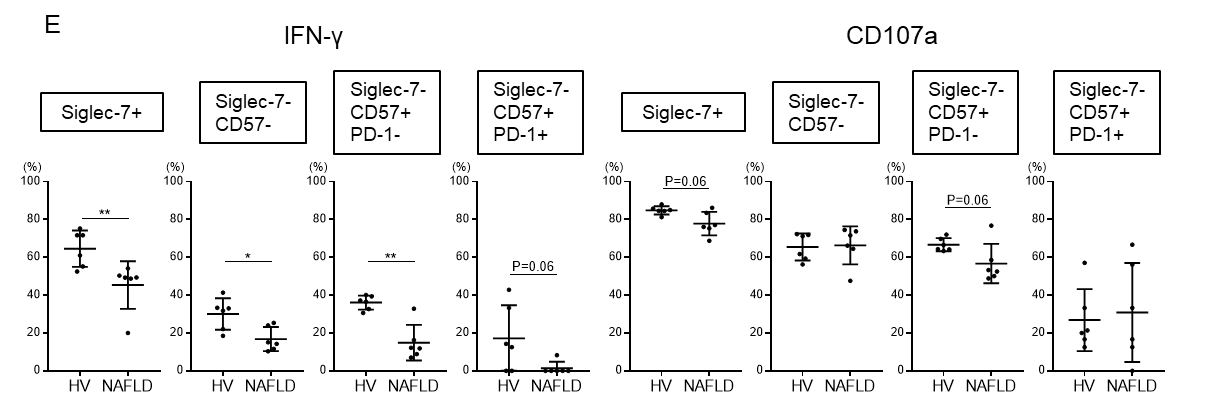
**

**Figure S4. Expression of Siglec-7 and CD57 on CD56^bright^ NK cells**

(A, B) Representative mass cytometry plots showing expression of Siglec-7 and CD57 on CD56^bright^ NK cells from HVs and NAFLD patients. (C) Percentage of Siglec-7^−^CD57^+^ within the CD56^bright^ NK cell subset from HVs (n=13) and NAFLD patients (n=42). (D) Frequency of Siglec-7^−^CD57^+^ NK cells within the CD56^bright^ and CD56^dim^ NK cell subpopulations. (E) Percentage of IFN-γ and CD107a expression on Siglec7^-^CD56^dim^ and Siglec7^+^CD56^dim^ subsets from HVs and NAFLD patients. Data are presented as the means ± SD (n=42) with individual patients represented by circles. ***P* < 0.01, *****P* < 0.0001 by the Mann–Whitney U-test (C), (E), and by the paired t-test (D). HV, healthy volunteer; NAFLD, non-alcoholic fatty liver disease; Siglec, sialic acid-binding immunoglobulin-like lectin; Sig7, Siglec-7

## Supplementary Figure 5

**
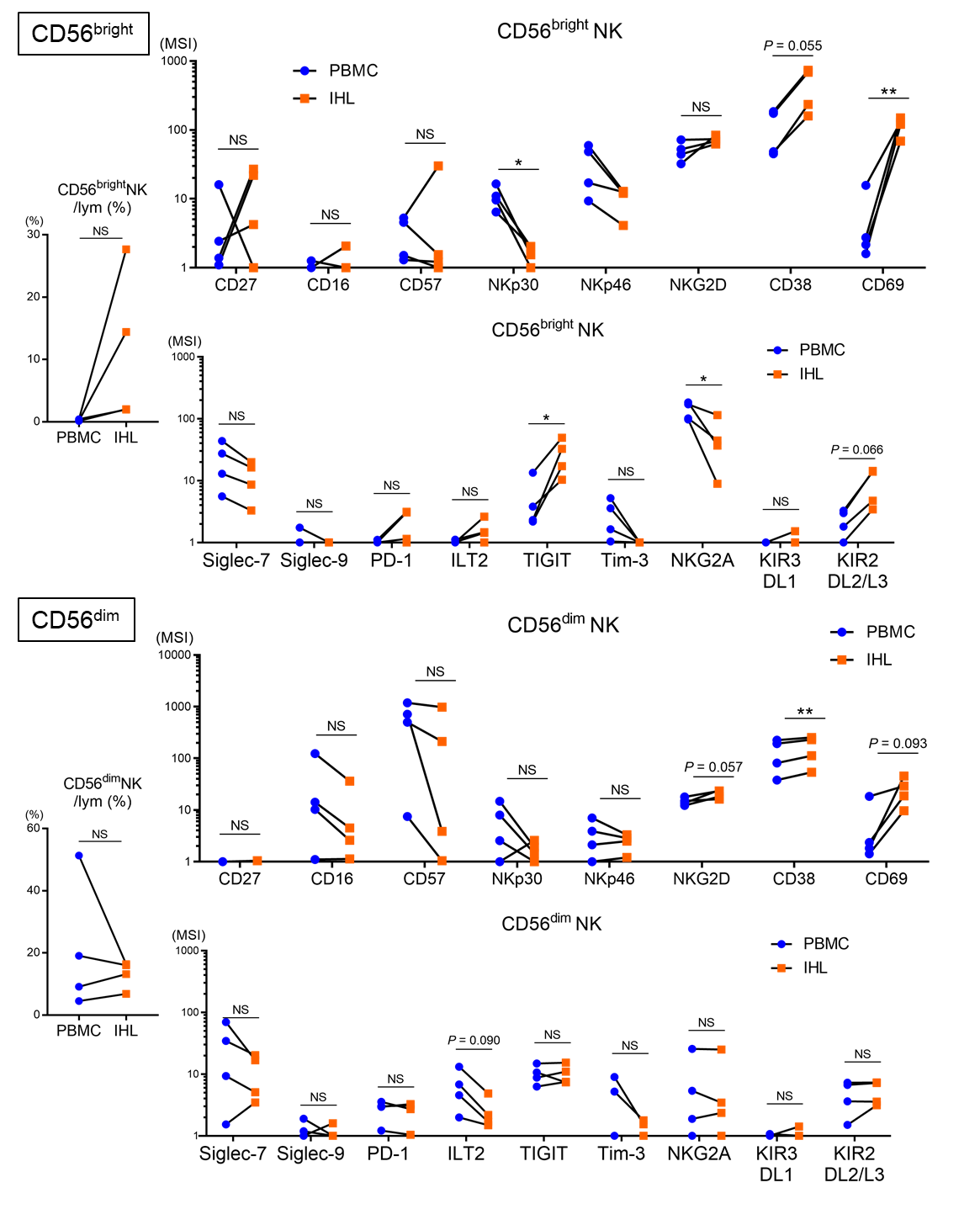
**

**Figure S5. Phenotype of NK cells in peripheral blood and non-cancerous liver tissues from patients with NAFLD-related HCC**

Expression levels (MSI) of 17 surface markers on CD56^bright^ and CD56^dim^ NK cell subpopulations of PBMCs (blue) and IHLs from non-cancerous liver specimens (orange) from patients with NAFLD-related HCC (n=4). Symbols represent the individual patients. *P* values were assessed by the paired t-test. IHL, intrahepatic lymphocyte; ILT2, immunoglobulin-like transcript 2; KIR2DL2/L3, killer cell immunoglobulin-like receptor 2DL2/L3; KIR3DL1, killer cell immunoglobulin-like receptor, three Ig domains and long cytoplasmic tail 1; lym, lymphocytes; MSI, median signal intensity; NAFLD, non-alcoholic fatty liver disease; NKG2A, CD94/NK group 2 member A; NKG2D, CD94/NK group 2 family of C-type lectin-like receptors; NS, not significant; PBMC, peripheral blood mononuclear cells; PD-1, programmed cell death-1; Siglec, sialic acid-binding immunoglobulin-like lectin; TIGIT, T-cell immunoreceptor with Ig and ITIM domains; Tim-3, T-cell immunoglobulin and mucin domain 3.

## Supplementary Figure 6

**
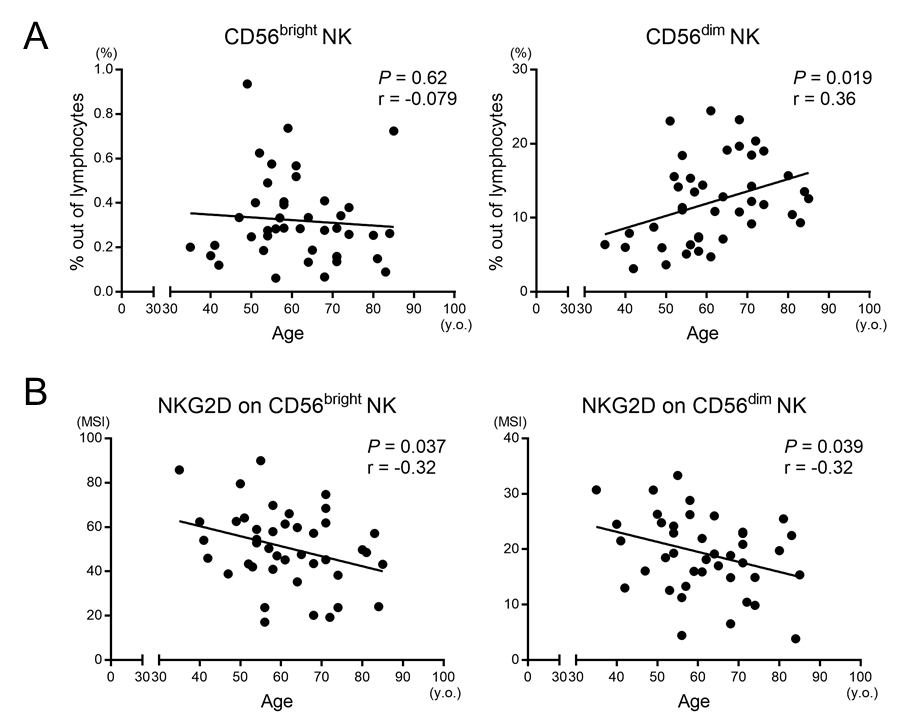
**

**Figure S6. Correlations between patient age and frequency of NK cell subsets or NKG2D expression in NAFLD patients**

Correlations between age and (A) percentage peripheral blood CD56^bright^ or CD56^dim^ NK cells and (B) NKG2D expression level (MSI) on CD56^bright^ or CD56^dim^ NK cells from NAFLD patients (n=42). *P* values and correlation coefficients (r) were calculated with Spearman’s correlation test. MSI, median signal intensity; NAFLD, non-alcoholic fatty liver disease; NKG2D, CD94/NK group 2 family of C-type lectin-like receptors.

## Supplementary Figure 7

**
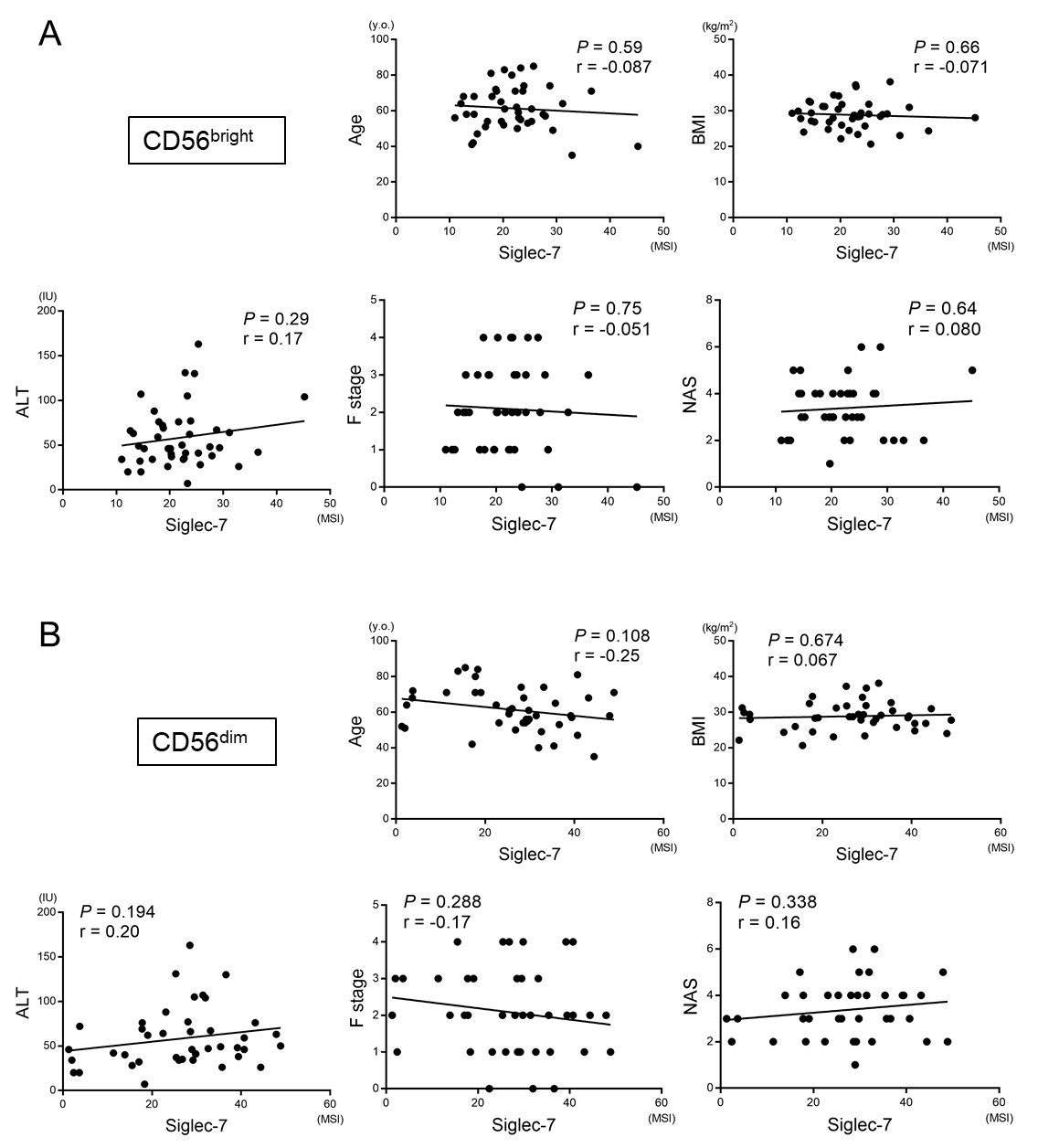
**

**Figure S7. Correlations between Siglec-7 expression on NK cells from NAFLD patients and clinical parameters**

Correlations between age, BMI, ALT, F stage, and NAS and (A) Siglec-7 expression on CD56^bright^ NK cells (A) and (B) Siglec-7 expression on CD56^dim^ NK cells (B) from NAFLD patients (n=42). *P* values and correlation coefficients (r) were calculated with Spearman’s correlation test. ALT, alanine aminotransferase; BMI, body mass index; F stage, fibrosis stage; MSI, median signal intensity; NAFLD, non-alcoholic fatty liver disease; NAS, NAFLD activity score; Siglec, sialic acid-binding immunoglobulin-like lectin; y.o., years old.

## Supplementary Figure 8


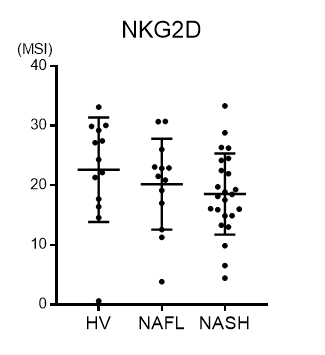


**Figure S8. Comparison of NKG2D expression on peripheral CD56^dim^ NK cells among healthy volunteers, NAFL, and NASH**

Expression levels (MSI) of NKG2D on CD56^dim^ NK cells from HVs (n=13), NAFL patients (n=13), and NASH patients (n=24) are shown. The diagnosis of NASH was determined by the classification of Matteoni. HV, healthy volunteer; MSI, median signal intensity; NAFL, non-alcoholic fatty liver; NASH, non-alcoholic steatohepatitis; NKG2D, CD94/NK group 2 family of C-type lectin-like receptors.
